# Phylogenetic Analysis of *Bacillus cereus sensu lato* Isolates from Commercial Bee Pollen Using tRNA^Cys^-PCR

**DOI:** 10.3390/microorganisms8040524

**Published:** 2020-04-06

**Authors:** José Luis Hernández Flores, Diana Salinas Landaverde, Yonuen Pacheco Huerta, Vania Lizeth Guerra Castillo, María de los Ángeles Barrios Sánchez, Iván Arvizu Hernández, Miguel Ángel Ramos López, Erika Álvarez Hidalgo, George H. Jones, Juan Campos Guillén

**Affiliations:** 1 Laboratorio de Bioseguridad y Análisis de Riesgo, Departamento de Ingeniería Genética, Centro de Investigación y de Estudios Avanzados del IPN, Irapuato 36824, Mexico; 2 Facultad de Química, Universidad Autónoma de Querétaro, Cerro de las Campanas S/N, Querétaro 76010, Mexico; dianasalinasl.97@gmail.com (D.S.L.); yonuenph1@gmail.com (Y.P.H.); vania.liz.17@live.com (V.L.G.C.); marye_1974@hotmail.com (M.d.l. Á.B.S.); iarvizu.her@icloud.com (I.A.H.); agromyke@gmail.com (M.Á.R.L.); erialvarez@yahoo.com (E.Á.H.); 3 Department of Biology, Emory University, Atlanta, GA 30322, USA; ghjones@emory.edu

**Keywords:** *Bacillus cereus*, *Bacillus thuringiensis*, *Bacillus bombysepticus*, tRNA^Cys^-PCR, MALDI-TOF MS, bee pollen

## Abstract

Endospore-forming bacteria related to the *Bacillus cereus* group produce toxins that cause illnesses in organisms from invertebrates to mammals, including foodborne illnesses in humans. As commercial bee pollen can be contaminated with these bacteria, a comprehensive microbiological risk assessment of commercial bee pollen must be incorporated into the relevant regulatory requirements, including those that apply in Mexico. To facilitate detection of members of this group of bacteria, we have developed a PCR strategy that is based on the amplification of the single-copy tRNA^Cys^ gene and specific genes associated with tRNA^Cys^ to detect *Bacillus cereus sensu lato* (*B. cereus s.l.*). This tRNA^Cys^-PCR-based approach was used to examine commercial bee pollen for endospore-forming bacteria. Our analysis revealed that 3% of the endospore-forming colonies isolated from a commercial source of bee pollen were related to *B. cereus s.l.,* and this result was corroborated by phylogenetic analysis, bacterial identification via MALDI-TOF MS, and detection of enterotoxin genes encoding the HBL and NHE complexes. The results show that the isolated colonies are closely related phylogenetically to *B. cereus, B. thuringiensis*, and *B. bombysepticus*. Our results indicate that the tRNA^Cys^-PCR, combined with other molecular tools, will be a useful approach for identifying *B. cereus s.l.* and will assist in controlling the spread of potential pathogens.

## 1. Introduction

Commercial bee pollen is a valuable natural product which, because of its nutrient composition, is considered a beneficial human foodstuff [1]. On the other hand, bee pollen can serve as a vector for the dissemination of endospore-forming bacteria through its use as a foodstuff by humans or in the rearing of insects of agriculture interest, or as an additive to promote animal growth [2,3,4,5,6,7]. Thus, preparations of commercial bee pollen must be tested for the presence of endospore-forming bacteria, such as *Bacillus cereus sensu stricto* (*B. cereus s.s.*) and regulations should be put in place that require such testing.

Furthermore, it is important to note that *B. cereus s.s.* is a member of the *Bacillus cereus sensu lato* group (*B. cereus s.l.*). This group contains an ecologically diverse collection of Gram-positive endospore-forming bacteria that are widespread in the environment and possess diverse metabolic capabilities that may play essential roles in various human activities [8,9,10]. The *B. cereus s.l.* group is a complex of organisms that are closely related phylogenetically and includes *B. anthracis*, *B. cereus s.s*, *B. thuringiensis*, *B. mycoides, B. pseudomycoides, B. weihenstephanensis, B. toyonensis, B. cytotoxicus*, *B. cereus* biovar *anthracis*, *B. gaemokensis*, *B. manliponensis*, *B. bingmayongensis*, and *B. wiedmannii* [11,12].

Decades of efforts to characterize and understand the taxonomic diversity of the *B. cereus s.l.* group have resulted in the development of a number of phenotypic and genetic methodologies for this purpose. For example, a number of features were used to establish Group I of this collection of microorganisms, including the morphology of the spore and sporangium, which produce central or terminal, ellipsoidal, or cylindrical spores that do not distend the sporangium [10]. Other features include the inability to produce acid from mannitol, the production of extracellular phospholipase (lecithinase), and the observation that these organisms are usually catalase-positive and are able to ferment a number of different carbohydrates [9,10]. Other features complicate the phenotypic differentiation of this group such as the observation that they may be aerobic or facultatively anaerobic, grow over a large temperature range, and may be motile via peritrichous flagellae (*B. cereus, B. cereus* var. *anthracis*, and *B. thuringiensis*) [9,10]. While *B. cereus*, *B thuringiensis*, and *B. mycoides* have been reported to be hemolytic and penicillin-resistant, *B. anthracis* is distinguished by its lysis by gamma phage [10,13].

Comparative genome analysis of members of this group of organisms [14] has revealed a close relationship between *B. anthracis*, *B. cereus*, and *B. thuringiensis,* suggesting that they may constitute a single species [15]. Other studies suggest that *B. mycoides and B. weihenstephanensis* are closely related species [16]. It is apparent that not all related species in the *B. cereus* group have the same capacity to induce disease in susceptible organisms, and this observation may be explained in part by the role of genetic determinants, such as plasmids, in the development of disease and in determining host specificity [17]. For example, the principal virulence factors in *B. anthracis* are encoded by genes located on two plasmids (pX01 and pX02). Similarly, the genes for crystal proteins in *B. thuringiensis* are located on plasmids. In contrast, in *B. cereus*, the virulence genes are chromosomally located and some related species share plasmids related to pX01 [18,19,20,21]. These plasmids can easily be transferred between these species so that the presence of plasmids in particular species is not a useful criterion for typing purposes.

Among the approaches used for the classification and taxonomic differentiation of *B. anthracis*, *B. cereus*, and *B. thuringiensis* are: 16S or 23S rRNA sequences; multilocus enzyme electrophoresis (MLEE); multilocus sequence typing (MLST); fluorescence amplified fragment length polymorphism analysis (AFLP) [22,23,24]; detection and analysis of toxin genes; and others [10]. However, none of these methods leads to conclusive identification of members of the *B. cereus* group and there continues to be some controversy over their applicability to this purpose [22,23,24].

Thus, while these phenotypic and molecular strategies for detecting *B. cereus s.l.* have their applications, their limitations and the fact that Mexico imports commercial bee pollen from a number of different countries led us to consider alternative molecular approaches for the identification of *B. cereus s.l*. In this study, we extend the observation previously reported for some tRNAs, such as tRNA^Cys^, which is encoded by a single-copy gene in some *Firmicutes* [25]. The position of this gene and its association with rRNA and tRNA operons or constitutive genes in the *B. cereus* genome recommend their use in molecular approaches for *B. cereus s.l.* detection. We report here the detection, based on tRNA^Cys^-PCR amplification in endospore-forming bacteria, of members of the *B. cereus s.l.* group obtained from commercial bee pollen and analyzed as a first step of colony selection based on genomic sequences.

## 2. Materials and Methods

### 2.1. B. cereus s.l. Detection

In Mexico, the best-known primary product of beekeeping is honey. Therefore, to satisfy the demand for bee pollen, significant amounts of pollen are imported into Mexico from other countries, such as Spain, China, Australia, and Argentina [25]. In the studies reported below, we analyzed pollen samples obtained from Europe via a commercial supplier. The specific country of origin of the pollen was not provided by the supplier. We analyzed six samples of bee pollen as the source of endospore-forming bacteria. Each replicate of 1 g of sample was homogenized in 10 mL of peptone and treated at 80 °C for 10 min to select endospore-forming bacteria. Serial dilutions were then inoculated into tryptic soy agar (TSA) medium (Difco Laboratories; Detroit, MI. USA) and incubated at 37 °C during 48 h. Numbers of spore-forming mesophilic bacteria (SMB) from each sample were determined and an average of 3.8 ± 0.03 log cfu/g was observed. Two hundred colonies obtained from the six samples that grew consistently under our conditions and manifested different phenotypic traits were tested by tRNA^Cys^-PCR.

Additionally, bacterial colonies were analyzed by the MALDI Biotyper as a microbial identification system based on MALDI-TOF mass spectrometry using a MicroFlex LT mass spectrometer (Bruker Daltonics) for species identification. The MALDI-TOF mass spectrometry method uses colonies directly after their treatment with 2 µl of MALDI matrix (a saturated solution of α-cyano-4-hydroxycinnamic acid in 50% acetonitrile and 2.5% trifluoroacetic acid). Spectra were analyzed by using the Bruker Biotyper 3.1 software (Bruker Daltonics). The identification score criteria used were those recommended by the manufacturer: a score ≥2.0 indicated species-level identification, a score between 1.7 and 1.9 indicated identification at the genus level, and a score <1.7 was interpreted as no identification.

### 2.2. tRNA Sequences Analysis

The tRNA sequences used in our analysis were aligned and extracted from complete *Bacillus* spp. genomes using the tRNAscan-SE program [26]. From predicted genes, tRNA counts per iso-acceptor were obtained for each genomic sequence and cluster analyses were done using the software R, version 3.0.2. The Kyoto Encyclopedia of Genes and Genomes (KEGG) database was used for tRNA^Cys^ gene region analysis for *Bacillus cereus* group genomes and some *Bacillus* species. The sequences from these regions were obtained and analyzed in MEGA X, using the Neighbor-Joining method [27,28,29,30]. The bootstrap consensus tree inferred from 1000 replicates was taken to represent the evolutionary history of the taxa analyzed.

### 2.3. Amplification Conditions

PCR primers were designed to amplify the tRNA^Cys^ region in the *Bacillus cereus* group. Primer 1517 (5’-GGCGGCATAGCCAAGTGGTAAGGC-3′) was designed to target the tRNA^Cys^ gene, while primer 1518 (5´- GCTGCCACATAAATTTCACGCCC-3′) was designed to target the *yebC*/*pmpR*-like gene. The *yebC*/*pmpR*-like and tRNA^Cys^ genes are located relatively close to each other. The primers were predicted to yield a product of 1145 bp. For tRNA^Cys^-PCR, *Bacillus cereus* ATCC 10876 (control) and a single colony (or in some cases, a pool of several colonies) of each bacterial strain tested were suspended in 50 µL of distilled water and heated to 95 °C for 10 min and 1 µL of the bacterial suspension was used in a 30 µL PCR mix using Phusion high-fidelity DNA polymerase (Thermo Scientific; Waltham, MA, USA). PCR conditions were as follows: a hot start (2 min, 94 °C), 35 cycles at 94 °C for 60 s, 55 °C for 30 s, and 72 °C for 30 s, final elongation step at 72 °C for 1 min. PCR products (5 µL) were analyzed by electrophoresis in 1.0% agarose (Sigma-Aldrich; St. Louis, MO, USA) and visualized on a UV transilluminator. Amplicons were sequenced using the platform at Macrogen Inc. (Seoul, Republic of Korea). Alignment and editing of gene sequences obtained were performed using MEGA X and compared with sequences of the *B. cereus* group by BLAST search in the NCBI, GenBank database (http://www.ncbi.nlm.nih.gov) and representative strains were selected for the analysis in MEGA X, using the Neighbor-Joining method [26,27,28,29,30]. The bootstrap consensus tree inferred from 1000 replicates was taken to represent the evolutionary history of the taxa analyzed. Branches corresponding to partitions reproduced in less than 50% of the bootstrap replicates were collapsed. All strains that tested positive in the tRNA^Cys^-PCR were evaluated for the presence of enterotoxin genes (*hblADC* and *nheABC*) with primers designed previously [31]. All positive strains were treated as above and amplified by PCR, and PCR conditions were as follows: a hot start (2 min, 94 °C), 35 cycles at 94 °C for 60 s, 50 °C for 30 s, and 72 °C for 30 s, final elongation step at 72 °C for 1 min. PCR products (5 µL) were analyzed by electrophoresis in 1.0% agarose (Sigma-Aldrich; St. Louis, MO, USA) and visualized on a UV transilluminator.

## 3. Results

### 3.1. Analysis of the tRNA Genes in B. cereus s.l. and Some Bacillus Species

**Figure 1:**
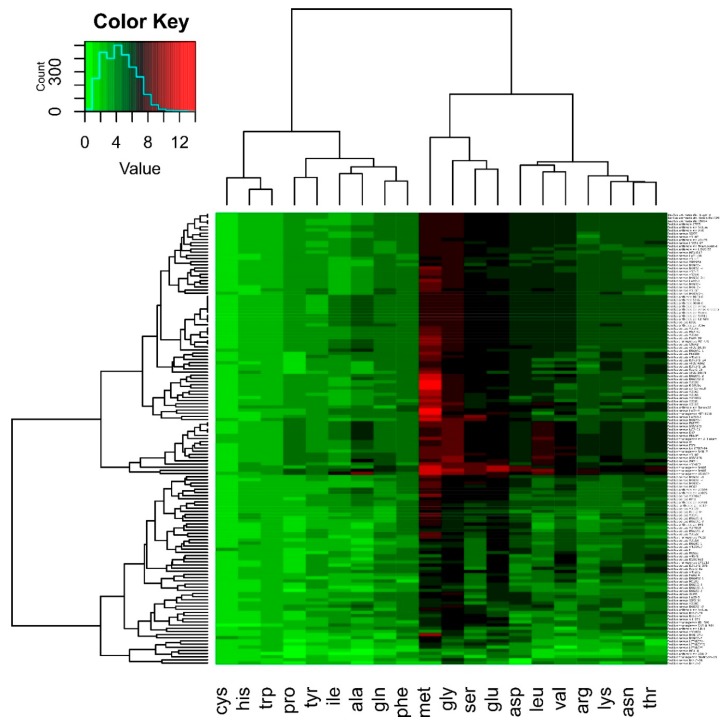
Distribution and copy number of tRNA genes in genomes of *Bacillus* spp. The tRNA genes were mapped and extracted using the tRNAscan-SE program. From predicted genes, tRNA counts per iso-acceptor were obtained and cluster analyses were done using the software R package, version 3.0.2. The Color Key refers to numbers of copies of the tRNA genes.

In this study, we extend our tRNA^Cys^-PCR method previously reported [25] to detect the presence of *B. cereus s.l.* in commercial bee pollen samples. As a first step, tRNA gene counts were predicted using tRNAscan-SE and compared with sequence data from *Bacillus* species. The results obtained in Figure 1 show that some tRNA iso-acceptors displayed relatively low copy numbers consistently across all genomes analyzed, such as tRNA^Cys^, tRNA^His^, and tRNA^Trp^. These tRNAs are represented by a light green color in Figure 1. Other tRNAs displayed relatively high copy numbers consistently across all genomes analyzed, such as tRNA^Met^, tRNA^Gly^, tRNA^Ser^, and tRNA^Glu^, and these are represented by black and red colors in Figure 1. Particularly relevant to this study, the tRNA^Cys^ gene was present in *B. cereus s.l.* in single copy. There were exceptions to this observation, as in *B. thuringiensis* or *B. pseudomycoides*-related species, where a second copy of tRNA^Cys^ is associated with genes that encode proteins, or in *B. megaterium*, where two copies are in tRNA clusters, while a third copy of tRNA^Cys^ is associated with genes that encode proteins. Based on these observations, we concluded that it would be valid to use the tRNA^Cys^ gene to develop a diagnostic method for *B. cereus s.l.* detection.

**Figure 2:**
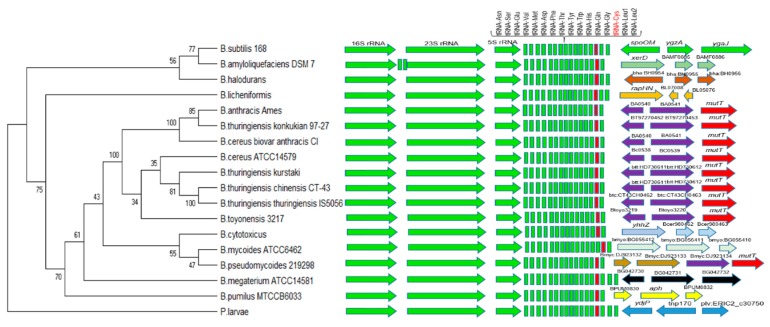
tRNA^Cys^ gene region analysis for the *Bacillus cereus* group and some related species. 16S, 23S, 5S rRNA, and the cluster of 15 to 17 tRNA genes found downstream of the 5S rRNA gene are shown. tRNA^Cys^ is in red color in the tRNA gene cluster and the orientation of three or four specific genes located downstream of tRNA^Cys^ is shown in various other colors. For our PCR strategy, we used the second *yebC*/*pmpR* gene (which encodes a probable transcriptional regulatory protein) located downstream from tRNA^Cys^ and between the first gene sequence (protein of unknown function) and the *mutT*-like gene (7,8-dihydro-8-oxoguanine-triphosphatase) indicated with purple and red colors, respectively. The sequences from these regions were obtained and analyzed in MEGA X, using the Neighbor-Joining method. *Paenibacillus larvae* was designated as the outgroup taxon.

The analysis of the tRNA^Cys^ gene in *B. cereus s.l.* and some *Bacillus* species not related to this bacterial group show that it is located at the distal end of a ribosomal operon, which codes for three rRNA molecules, 16S, 23S, and 5S rRNA, and a cluster of 15 to 17 tRNA genes found downstream of the 5S rRNA gene (Figure 2). For the development of our PCR strategy, we used the second *yebC*/*pmpR*-like gene (which encodes a probable transcriptional regulatory protein) located downstream from tRNA^Cys^ and between the first gene sequence (protein of unknown function) and a *mutT*-like gene (7,8-dihydro-8-oxoguanine-triphosphatase) indicated in Figure 2. After the alignment of the selected sequences, those sequences were found to be specific for *B. anthracis*, *B. cereus s.s.*, *B thuringiensis*, and *B. toyonensis*-related species and were therefore suitable for the design of specific primers.

*B. mycoides, B. pseudomycoides*, and *B. cytotoxicus* show a different gene organization than do several of the other species analyzed in Figure 2, similarly to some *Bacillus* species not related to this bacterial group. In *B. cytotoxicus*, a gene located downstream from tRNA^Cys^ encodes an HNH endonuclease, and that gene is followed by two others that encode a SMI1/KNR4 family protein (SUKH-1). In *B. mycoides*, an additional tRNA^Met^ is located in the cluster of tRNA genes and a sequence located downstream from tRNA^Cys^ encodes a phage integrase family protein, followed by two genes of unknown function. In *B. pseudomycoides,* a sequence located downstream from tRNA^Cys^ encodes an integrase/recombinase domain protein, followed by a sequence that encodes a papain cysteine protease family protein and two sequences similar to YebC and mutT family proteins. Additionally, in the cluster of tRNA genes, *B. subtilis* and *B. licheniformis* have two tRNA^Leu^ genes downstream from tRNA^Cys^, while *B. halodurans* has a tRNA^Arg^ instead of tRNA^Leu^ in the last position. *B. megaterium* presents two tRNA^Leu^ genes and one tRNA^Gly^ gene downstream from tRNA^Cys^. *Paenibacillus larvae* was designated as the outgroup taxon and presents a tRNA^Arg^ among two tRNA^Leu^ genes at the end of the cluster of tRNA genes.

### 3.2. B. cereus s.l. Detection from Colony

**Figure 3:**
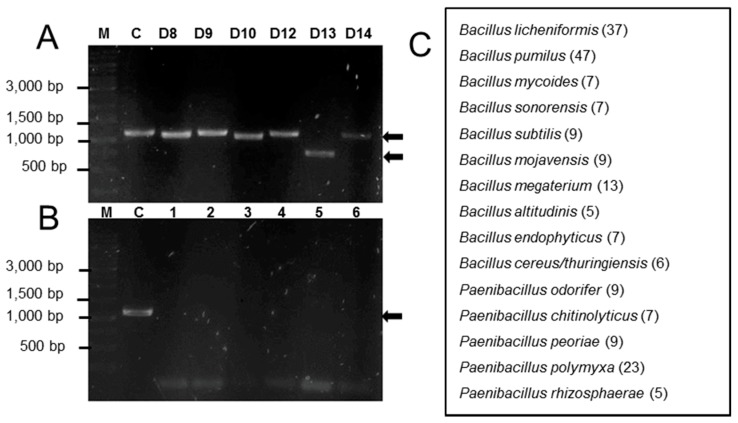
*B. cereus s.l.* detection in commercial bee pollen. (**A**) Panel A show positive results of the tRNA^Cys^-PCR analyses for isolates D8 to D14 and (**B**) panel B show negative results for tRNA^Cys^-PCR (see text for bacterial species identified for lanes 1 to 6). Lane M contains a Thermo Scientific GeneRuler 1 Kb DNA ladder, lane C is the control (*B. cereus* ATCC 10876), and the arrows show the PCR amplification product. (**C**) Bacterial species identified by MALDI-TOF MS; numbers in parentheses indicate the total number of isolated colonies identified.

To test the utility of the tRNA^Cys^-PCR strategy for the detection of *B. cereus s.l.* in bacterial colonies obtained from commercial bee pollen, we recovered 200 colonies of endospore-forming bacteria that grew consistently on TSA medium under our conditions, and analyzed them by tRNA^Cys^-PCR as described in Materials and Methods. From these colonies, only six (3%) were positive in the tRNA^Cys^-PCR, and electrophoresis of the products showed at least three different PCR amplification sizes (Figure 3A). For the bacterial colonies indicated as D9, D12, and D14 in Figure 3A, the PCR product had the same mobility as that from the control species (*B. cereus* ATCC 10876), while for bacterial colonies D8 and D10, the PCR product migrated slightly ahead of the control. Interestingly, a PCR product of around 750 bp was detected for the bacterial colony D13.

To determine the validity of the tRNA^Cys^-PCR positive (Figure 3A) and negative (Figure 3B) results, all the bacterial colonies analyzed by PCR were also identified by MALDI-TOF MS following the score criteria described in Materials and Methods (Figure 3C). This analysis identified members of the genus *Bacillus* (147 isolated colonies) as the most common species found in association with commercial bee pollen, followed by *Paenibacillus* species (53 isolated colonies). We also confirmed the positive results of the tRNA^Cys^-PCR analyses for isolates D8 to D14 (Figure 3A) by MALDI-TOF MS. That analysis also identified these isolates as *Bacillus cereus/thuringiensis*. Figure 3B shows results from the tRNA^Cys^-PCR analysis that did not produce an amplification product. These isolates were identified by MALDI-TOF MS as *B. endophyticus* (lane 1), *B. subtilis* (lane 2), *B. altitudinalis* (lane 3), *B. sonorensis* (lane 4), *P. odorifer* (lane 5), and *P. polymyxa* (lane 6).

**Figure 4:**
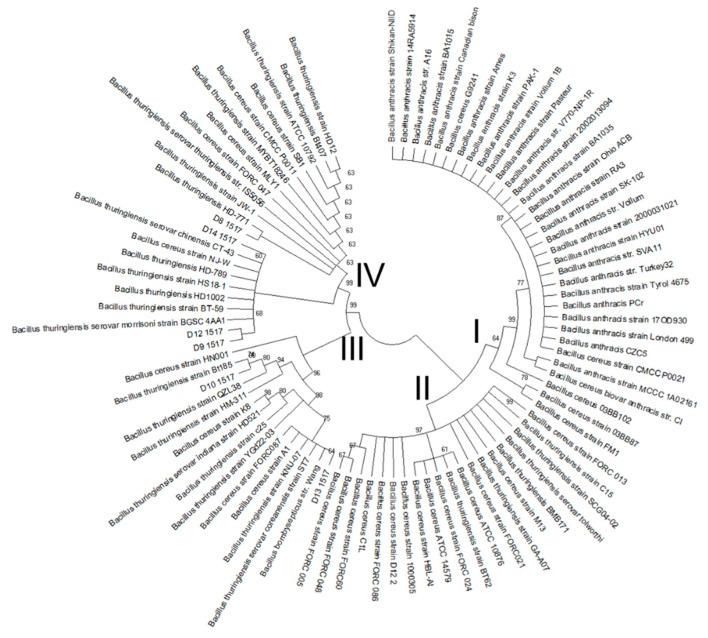
Phylogenetic analysis in MEGA X, using the Neighbor-Joining method. tRNA^Cys^- *yebC*/*pmpR* genes sequences of the isolates were compared with sequences of *B. cereus* group obtained by BLAST search in the NCBI, GenBank database (http://www.ncbi.nlm.nih.gov) and representative strains were selected for the analysis. The bootstrap consensus tree inferred from 1000 replicates is taken to represent the evolutionary history of the taxa analyzed. Branches corresponding to partitions reproduced in less than 50% of the bootstrap replicates are collapsed. Group I contained almost exclusively species that were closely related to *B. anthracis*, while groups II, III, and IV contained species phylogenetically related to *B. cereus* and *B. thuringiensis*. The isolated colonies obtained are shown as D8 1517, D9 1517, D10 1517, D12 1517, D13 1517, and D14 1517 appearing within groups III and IV.

**Figure 5:**
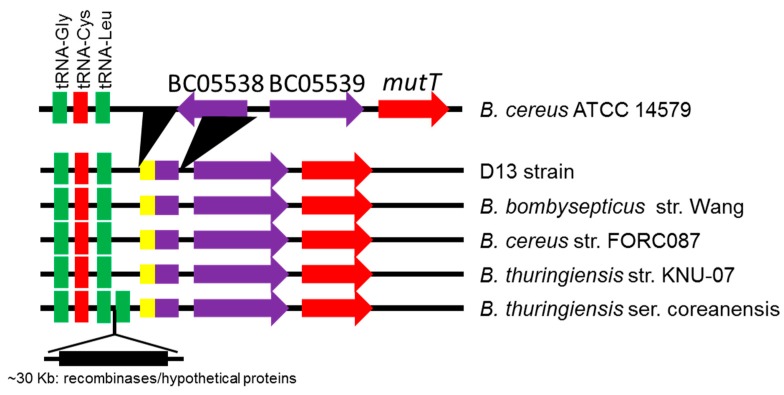
Analysis of the tRNA^Cys^ region for strain D13 and species closely related phylogenetically. Deletion of sequences is indicated by black triangles compared with *B. cereus* ATCC 14579. Insertion of ~30 Kb for *B. thuringiensis* ser. coreanensis is indicated by the black rectangle. The yellow color indicates insertion sequences detected.

Our phylogenetic analysis confirmed the results obtained by tRNA^Cys^-PCR and MALDI-TOF MS, viz. that the bacterial colonies identified by tRNA^Cys^-PCR are members of the *B. cereus* group. Clustering of the tRNA^Cys^- *yebC*/*pmpR* genes sequences revealed four major species groups (Figure 4). Group I contained almost exclusively species that were closely related to *B. anthracis*, while groups II, III, and IV contained species phylogenetically related to *B. cereus* and *B. thuringiensis.* The species isolated from commercial bee pollen were contained in Groups III and IV, closely related phylogenetically to *B. thuringiensis* and *B. cereus* strains from diverse environmental sources. When we analyzed the phylogenetic results for the isolate D13 (group III), which presented a shorter PCR product, we obtained a surprising result. The DNA sequence showed a high similarity to *B. bombysepticus* strain Wang and some related species of *B. cereus* and *B. thuringiensis*, all isolated from China or South Korea (Figure 5). These phylogenetically related species show a deletion of around 132 nucleotides in the intergenic region between the tRNA cluster and the next gene located downstream, as well as a deletion of around 348 nucleotides that includes part of the first gene and the intergenic region before the second gene located downstream (the *yebC*/*pmpR* gene). The genomic analysis reveals that for *B. thuringiensis* serovar coreanensis there is an insertion of ~30 Kb in the intergenic region between the tRNA cluster and the next gene located downstream, with duplication of tRNA^Leu^ and loss of sequences similar to the phylogenetically related species (Figure 5). The presence of genes related to recombinases in this insertion suggests that it arose by a recombination event.

As a complement to our other analyses, the bee pollen bacterial isolates (colonies D8 to D14) were evaluated for the presence of six enterotoxic genes HBL (*hblA*, *hblD*, and *hblC*) and NHE (*nheA*, *nheB*, and *nheC*) by PCR. All strains tested yielded a PCR amplification product for each of the six genes (Figure S1). These results show that all these bacterial strains have the potential to synthetize toxins involved in human diarrheal disease, but the clinical relevance of this observation remains to be evaluated.

## 4. Discussion

In Mexico, the demand for bee pollen has grown during the last few decades, so that import from countries such as Spain, China, Australia, and Argentina is necessary to satisfy that demand [32]. Because of differences in geography, flora, and production methods, from a biosecurity perspective, bee pollen could be exposed to microbial contamination during its production, storage, or commercialization. Therefore, quality and safety must be important considerations in certifying commercial bee pollen for use, with the specific challenge of identifying potential pathogenic species in pollen preparations [2,5,6,33,34].

However, based on our knowledge of the situation in Mexico and in many other countries, microbial studies of commercial bee pollen do not include detection of endospore-forming bacteria with the potential to produce disease-causing toxins [32]. Indeed, regulatory requirements are in general not well defined or do not include detailed instructions for microbial testing of imported pollen shipments. These deficiencies could contribute to the spread of potential pathogenic species and cause food-borne illnesses in organisms for which bee pollen is designated for feed consumption [2,3,4,5,6,7].

The studies presented here demonstrate for the first time the presence in commercial bee pollen of bacterial species closely related phylogenetically to the *B. cereus* group based on tRNA^Cys^-*yebC*/*pmpR* genes sequences, but also the presence of other species identified by MALDI-TOF MS from the genera *Bacillus* and *Paenibacillus*. These last species could be a promising source of strains producing metabolites with antimicrobial activity, which has been investigated by other authors [35,36]. Moreover, our studies demonstrate the presence of enterotoxin genes (*hblADC* and *nheABC*) in the colony isolates D8 to D14, which could increase the potential for these strains to cause food-borne illnesses. These results should be of particular interest to the Mexican Agriculture Ministry, and to those of other countries, as they endeavor to control the quality of commercial bee pollen imports.

Although various sterilization methods are used to eliminate microorganisms present in pollen [37,38,39], a survival strategy available to *B. cereus s.l,* is that under unfavorable environmental conditions that affect cell growth, the organism coordinates a temporal molecular program to assemble a dormant spore. This spore is highly organized within a dehydrated spore core, which is enveloped by a dense inner membrane, a germ cell wall, a cortex, and a spore coat [40]. Each spore component is formed by molecules that play major roles in protecting the spore from a broad range of detrimental environmental conditions such as radiation, heat, desiccation, and chemicals [41,42,43,44]. These spore characteristics allow for survival during pollen processing treatments and make it difficult to eradicate spores in bee pollen sources. There is, therefore, reason for concern about the possibility that imported commercial bee pollen may be contaminated with members of the *B. cereus* group, resulting in the potential risk of pathogenic outcomes from the use of that pollen.

Several *B. cereus* strains have been identified as opportunistic food-borne pathogens, and several different toxins have been associated with food poisoning outbreaks caused by these strains, viz. cereulide, cytotoxin K, hemolysin BL (HBL), and non-hemolytic enterotoxin (NHE) [10,13,17,45,46]. In this regard, and in accord with other reports, bacterial members of the *B. cereus* group have been isolated and identified using phenotypic characteristics or molecular approaches based on 16S rRNA or MALDI-TOFF MS in bee pollen obtained from hives or commercial sources [25,47,48]. Enterotoxin genes (*hblADC* and *nheABC*) were detected by PCR in our studies but it will be necessary to examine toxin production more carefully in these species in order to evaluate the potential for food-borne illnesses that might be caused by bee pollen consumption.

Based on tRNA^Cys^-*yebC*/*pmpR* genes sequences, our results show that the six isolated colonies (D8 to D14) are clustered within Groups III and IV of Figure 4, along with species closely related phylogenetically to *B. thuringiensis* and *B. cereus,* and separated from group I, where *B. anthracis* is predominant. An interesting outlier in our analysis was isolate D13, which shares a close relationship with *B. bombysepticus* strain Wang. This strain has been linked to *Bombyx mori* black chest septicemia [49], but additional comparative genomic studies of isolate D13 are necessary to understand its metabolic capabilities. Consistent with other studies demonstrating insertions and deletions in the genomes of various *B. cereus* strains [13,14,15,20,21], our studies provide evidence for recombination events leading to insertions and deletions which may be relevant both to the potential pathogenicity of these species as contaminants of bee pollen and to the evolutionary history of these lineages within the *B. cereus* group.

It must be noted that the existing literature suggests a high degree of genetic diversity between the various species examined here, and questions related to the taxonomy of *B. anthracis*, *B. cereus*, and *B. thuringiensis* continue to be argued among researchers in this field [9,10,11,12,13,14,15,16,17,18,19,20,21,22,23,24]. Additional insight into these taxonomic questions will no doubt be provided by comparative genomics, as well as by molecular discrimination analyses using prominent biomarkers such as the chaperonin protein (*GroEL*) and the topoisomerase (*gyrB*) [50]. In addition, a more thoroughgoing analysis of toxin production by the strains isolated in our study will address the question of the close relationship among members of the *B. cereus* group.

In conclusion, in order to detect the presence of *B. cereus s.l.* in commercial bee pollen samples and as a tool for controlling the spread of this potential pathogen, we used the tRNA^Cys^-PCR method, which could be a fast and straightforward approach that complements those that already exist for detection of this group of microbes. Moreover, this could be the method of choice when large numbers of endospore-forming bacterial colonies are to be screened as a first step to select *B. cereus s. l.* colonies.

## Supplementary Materials

The following are available online at https://www.mdpi.com/2076-2607/8/4/524/s1.
