# Supplementary material for: Phylogenetic Analysis of Bacillus cereus sensu lato Isolates from Commercial Bee Pollen Using tRNACys-PCR
Source: Microorganisms. 2020 Apr 6;8(4):524. doi: 10.3390/microorganisms8040524 (PMC7232370; doi:10.3390/microorganisms8040524)
Supplement: Supplementary file 1 [file microorganisms-08-00524-s001.pdf]

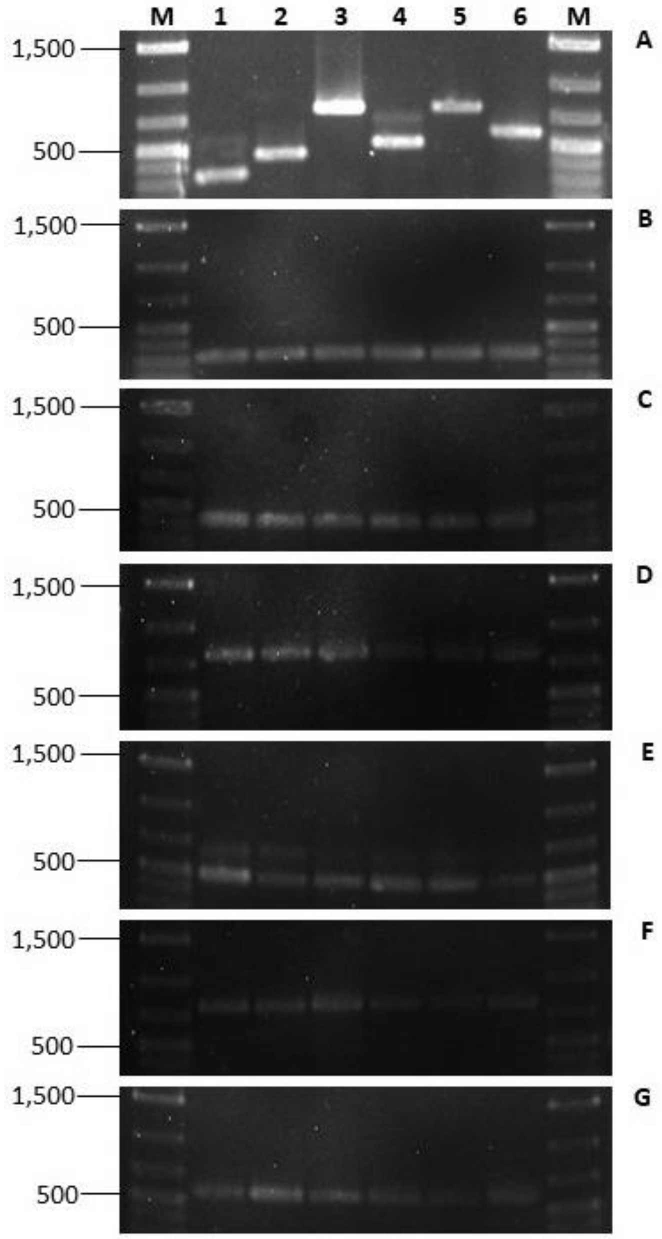

Figure S1. PCR results from isolated *B. cereus sensu lato*. Panel A, is the control (*Bacillus cereus* ATCC 10876) agarose gel of PCR products for the *hblA* (lane 1, 320 bp), *hblD* (lane 2, 430 bp), *hblC* (lane 3, 750 bp), *nheA* (lane 4, 500 bp), *nheB* (lane 5, 770 bp) and *nheC* (lane 6, 582 bp) encoding enterotoxic genes HBL and NHE. Panel B-D are PCR for *hblADC* and panel E-G for *nheABC* genes detected in isolated D8, D9, D10, D12, D13 and D14, respectively. Lane M contains a Thermo Scientific GeneRuler 1 Kb Plus DNA ladder.
